# A cost-utility analysis comparing endovascular coiling to neurosurgical clipping in the treatment of aneurysmal subarachnoid haemorrhage

**DOI:** 10.1007/s10143-022-01854-9

**Published:** 2022-09-03

**Authors:** Ayla Ahmed, Yonis Ahmed, Kwaku Duah-Asante, Abayomi Lawal, Zain Mohiaddin, Hasan Nawab, Alexis Tang, Brian Wang, George Miller, Johann Malawana

**Affiliations:** 1 Faculty of Medicine, Department of Medicine, Imperial College London, London, UK; 2 Department of Metabolism, Digestion and Reproduction, Imperial College Healthcare Trust, London, UK; 3 Centre for Digital Health and Education Research, School of Medicine, University of Central Lancashire, Preston, UK

**Keywords:** Endovascular coiling, Neurosurgical clipping, Aneurysmal subarachnoid haemorrhage, Cost-utility analysis, Economic evaluation

## Abstract

Endovascular coiling (EC) has been identified in systematic reviews and meta-analyses to produce more favourable clinical outcomes in comparison to neurosurgical clipping (NC) when surgically treating a subarachnoid haemorrhage from a ruptured aneurysm. Cost-effectiveness analyses between both interventions have been done, but no cost-utility analysis has yet been published. This systematic review aims to perform an economic analysis of the relative utility outcomes and costs from both treatments in the UK. A cost-utility analysis was performed from the perspective of the National Health Service (NHS), over a 1-year analytic horizon. Outcomes were obtained from the randomised International Subarachnoid Aneurysm Trial (ISAT) and measured in terms of the patient’s modified Rankin scale (mRS) grade, a 6-point disability scale that aims to quantify a patient’s functional outcome following a stroke. The mRS score was weighted against the Euro-QoL 5-dimension (EQ-5D), with each state assigned a weighted utility value which was then converted into quality-adjusted life years (QALYs). A sensitivity analysis using different utility dimensions was performed to identify any variation in incremental cost-effectiveness ratio (ICER) if different input variables were used. Costs were measured in pounds sterling (£) and discounted by 3.5% to 2020/2021 prices. The cost-utility analysis showed an ICER of − £144,004 incurred for every QALY gained when EC was utilised over NC. At NICE’s upper willingness-to-pay (WTP) threshold of £30,000, EC offered a monetary net benefit (MNB) of £7934.63 and health net benefit (HNB) of 0.264 higher than NC. At NICE’s lower WTP threshold of £20,000, EC offered an MNB of £7478.63 and HNB of 0.374 higher than NC. EC was found to be more ‘cost-effective’ than NC, with an ICER in the bottom right quadrant of the cost-effectiveness plane—indicating that it offers greater benefits at lower costs. This is supported by the ICER being below the NICE’s threshold of £20,000–£30,000 per QALY, and both MNB and HNB having positive values (> 0).

## Introduction

An acute subarachnoid haemorrhage (aSAH) is a medical emergency that accounts for 5% of all strokes worldwide [1] corresponding to an incidence of 9 per 100,000 person-years [1]. Approximately half of aSAH patients are below 55 years of age [2] and are affected by an especially high disease-specific burden and fatality rate, with a third of patients dying within the days or weeks afterward [2] and an overall 45% mortality within the first month [3].

Taken together, aSAH presents a vast socio-economic burden to the UK’s population and its healthcare system, the National Health Service (NHS). In 2005, aSAH caused a loss of approximately 80,356 life years and 74,807 quality-adjusted life years (QALYs) in the UK [4]. aSAH is estimated to cost the UK £510 million and the National Health Service (NHS) £168.2 million annually, with each patient costing £23,294 on average [4]. Eighty-five percent of aSAH cases are caused by aneurysmal rupture [5], while the remainder result from traumatic head injury. aSAH classically presents as a sudden onset severe headache, often described as a ‘thunderclap’, alongside a host of other symptoms [6]. Confirmation of diagnosis is via CT scan, with patients referred to specialist neurology services for treatment [6]. To prevent imminent complications such as secondary cerebral ischaemia, patients are given medications including pain relief, anticonvulsants, antiemetics and calcium channel blockers such as nimodipine [6]. A surgical treatment is then required to repair the site of bleeding and reduce risk of rebleeding [6]. The NHS currently offers two treatments for aneurysmal aSAH that are both performed under general anaesthetic [6]: EC and NC. Choice of procedure is often dependent on the size, shape and location of the aneurysm as well as a number of patient factors.

EC is a minimally invasive procedure involving the insertion of a catheter through the femoral artery [7]. Once guided to the brain, a platinum coil attached to the tip of the catheter is released at the lumen of the aneurysm. The coil is left in the aneurysm permanently, inducing thrombosis and thus occluding blood flow [7]. NC is a more traditional procedure, performed via craniotomy [8]. Once located, a titanium clip is positioned over the neck of the aneurysm to prevent any further blood flow through its lumen.

This study is a cost-utility analysis comparing the use of EC to NC in the treatment of a bleeding subarachnoid haemorrhage due to aneurysm rupture.

## Materials and methods

### Outcome measurement and choice of analysis

A cost-utility analysis (CUA) was carried out in this economic evaluation. CUA aims to compare the total costs and health effects of alternative interventions, to determine which intervention yields the highest utility for the associated costs. CUA is regularly performed by the National Institute for Health and Care Excellence (NICE) to inform decisions on treatment provisions in the NHS.

The costs associated with EC and NC along with the patient’s degree of disability post-surgery were measured in pound sterling (£s). Outcomes were measured in terms of the patient’s postoperative modified Rankin scale (mRS) grade, a widely used 6-point disability scale aiming to quantify a patient’s functional outcome following a stroke [9]. Each mRS health state is shown in Table 1.

The categorical nature of the scale, however, means the mRS score may not account for potentially unequal differences in perceived quality of life associated with certain 1-point changes compared to others [9]. For example, in assigning patients to categories ranging from 0 (no symptoms) to 6 (death), the mRS score does not account for patients who may prefer death over the mRS 5 health state (bedridden, incontinent and requiring constant assistance) [9]. To account for this, the Stroke Treatment Academic Industry Roundtable (STAIR VII) called for the development of a utility-weighted mRS (UW-mRS) that associates each mRS score with an established health utility scale [9]. Rebchuk et al. pooled together 24 studies exploring utility weightings for the mRS score, calculating average utility weights for each mRS score using both the time trade-off and person trade-off techniques [9]. In this study, the mRS score was weighed against the Euro-QoL 5-dimension (EQ-5D) [9].

The EQ-5D questionnaire is a widely accepted multi-attribute instrument used to assess health-related quality of life. The questionnaire requires participants to score themselves on either a 3- or 5-level scale based on severity of symptoms in five different domains: mobility, self-care, usual activities, pain/discomfort and anxiety/depression [10]. Assigning a utility weighting (EQ-5D) to each mRS state enables conversion to QALYs as an outcome measure. QALYs are a standardised measure of disease burden combining health-related quality of life with survival (or length of life) [11], reported as a number ranging from 0 (death) to 1 (no symptoms) [11]. The EQ-5D scale provides a utility score for each mRS health state, which can be multiplied with the length of time spent in this health state to convert to QALYs [10]. The time horizon for this study is 1 year; this was used as the length of time spent in the mRS state.

The conversion of the mRS score to QALYs using utility-based weightings calls for the conduction of a CUA. The outcome measure attempts to quantify quality of life achieved through EC compared to NC in the emergency treatment of an acute subarachnoid haemorrhage. A cost-effectiveness analysis will not be carried out as measuring outcomes solely in terms of mRS state does not account for differences in perceived quality of life between health states. Similarly, a cost–benefit analysis is not appropriate in this case owing to loss of accuracy in the conversion of outcome measures to monetary units.

In summary, we used mRS states as the outcome measure and assigned each state a utility based on the EQ-5D utility scale. We then converted these utility values to QALYs, which we then used to conduct our CUA.

### Costs used and justification

We broke down the costs into two main sources—the intervention cost and the treatment cost. The intervention cost refers to the cost of the procedure itself (EC or NC), while the treatment cost refers to the cost of patient care in the first 12 months following their procedure, depending on their resultant mRS state.

The intervention cost data on both EC and NC as treatment pathways for aneurysmal subarachnoid haemorrhage was obtained from a study performed on behalf of the ISAT collaborative group by Wolstenholme et al., which recorded the overall resource usage and cost of each treatment strategy [3]. The data was based on a sample of patients from ISAT and contained patients across 22 UK centres (*n* = 1644). Costs were expressed in Great British pounds (£) for the year 2004, after being inflated from the preceding years. Any costs incurred over subsequent 12- and 24-month periods were discounted at a rate of 3.5%.

The study from which we obtained intervention costs provided follow-up treatment costs; however, these costs were not categorised based on the primary clinical outcome (by mRS state). Therefore, only the intervention costs incurred from the first episode of care were used in the present study, which included the cost of intervention, imaging and investigations, and hospital stay (shown in Tables 2 and 3). The total cost per patient for EC and NC was £30,431 and £34,714, respectively.

The data on costs incurred based on each mRS health state (over 12 months) was obtained from a recent paper published in 2019 [12], identifying direct medical costs after stroke using mRS as the determinant [13]. As the mRS is a nominal indicator of health state following a stroke, naturally, it was found that higher (more severe disability) mRS scores were associated with higher costs as the level of care required increased. Post-intervention costs for each mRS score can be further divided into inpatient and outpatient costs. Inpatient care constitutes re-admission post-intervention, days in rehabilitation and days spent in a nursing home. Outpatient care can be categorised into further A&E visits, clinic appointments and rehabilitation therapies. Professionals involved in outpatient care include GPs, secondary care physicians such as neurologists, cardiologists and geriatricians, nurses, physiotherapists and speech therapists. A summary table detailing costs of treating each mRS state can be seen in Table 4.

### Discount rate and justification

The monetary costs and health benefits must first be converted to their ‘present value’ in order to account for the variation in value over time. Costs were discounted by 3.5% annually in accordance with NICE guidelines [14]. mRS treatment costs were originally obtained from a study done in 2016/2017, while intervention costs were obtained from a study done in 2003/2004. Thus, these costs were discounted by 4 and 17 years respectively to calculate 2020/2021 costs. Outcome data (QALYs) was not discounted, as our study assumed that regardless of time difference, the health benefits would retain equal value [15].

### Modelling

**Fig. 1:**
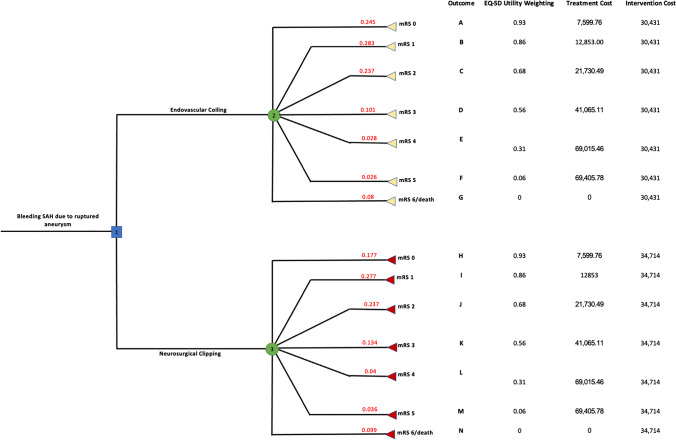
Decision tree detailing possible outcomes of the two treatment arms with associated utility weightings, complication treatment and intervention costs

Expected utility (EU) was measured in QALYs, and expected cost (EC) was measured in £s. The breakdown of probabilities for each mRS branch (clinical outcome) was provided by the ISAT trial [16] and can be seen in Table 5. These were then multiplied by utility values and costs of both the intervention itself as well as costs associated with each mRS health state, to calculate the total EU and EC as seen in our decision tree (Fig. 1).

The total EU calculated for nodes 2 and 3 is representative of the total weighted utility associated with EC and NC, respectively. Utility values assigned to each mRS state obtained from Rebchuk et al. were expressed in terms of the EQ-5D scale [9] and multiplied by the associated probability of each mRS state to calculate the utility for each branch of the tree. The EU for each node was then obtained by totalling the utility calculated for each of the 7 branches.

The cost assigned to each of the branches consisted of both the cost of the intervention itself (Tables 2 and 3) as well as the costs associated with each mRS state (Table 4). For example, a patient classed as mRS 5 post-EC represents a total cost of £38,031, with £30,431 being the intervention cost and £7600 the cost associated with treating the mRS stage. The standardised nature of the mRS score, utilised broadly in stroke clinical trials, allows for these same figures to be assigned in this study [17]. The same mRS costs were used in both treatment arms as patients in each state are assumed to consume similar levels of healthcare resources in the first year following surgery, regardless of the intervention undergone. The cost of the intervention alone was used for mRS 6, which represents death and therefore does not incur any healthcare costs. The total cost of each branch was multiplied by the probability associated with each mRS state for NC and EC [9]. Values for each mRS state were totalled to calculate the total EC for nodes 2 and 3, respectively (Table 6). Total EU and EC values were then used in the calculation of the incremental cost-effectiveness ratio (ICER), health net benefit (HNB) and monetary net benefit (MNB).

The two initial treatment arms branch off node 1, representative of the decision to adopt one intervention over another. Patients suffering an aSAH lack the consciousness and capacity to provide consent and therefore no ‘acceptance’ arm was included [18]. Additionally, ‘rejection’ of the intervention results in certain and immediate death and therefore no evidence was found for the construction of a rejection pathway [18]. All cost and utility figures used pertain to the 1-year time horizon specified in this study.

### Choice of perspective and justification

This economic evaluation was carried out from the perspective of the NHS. Since both interventions are offered by the NHS for the treatment of aSAH, a CUA would therefore provide valuable insight into whether adopting one intervention over the other offers greater utility to patients relative to costs incurred by the healthcare system. This can facilitate improved decision-making on the allocation of NHS’s monetary resources and reduce the current economic burden aSAH presents to the UK.

### Choice of analytic horizon and justification

We assessed the cost-utility of both interventions over 1 year following each treatment, as data regarding both outcome (mRS scores) [16] and costs was only retrievable for this time frame. However, we found that a 1-year analytic horizon was suitable as the majority of post-treatment procedures and follow-up treatments would occur within the first year [19], suggesting that most complications and hence costs, would be incurred within 12 months post-intervention. Additionally, the outcome is almost immediately following recovery from surgery, with patients assigned an mRS category representing their gained utility. Thus, standardising our economic evaluation to a 1-year time period would encapsulate most of the materialised costs and utilities of both interventions in order to generate a meaningful CUA.

## Results

### ICER

The ICER calculated represents the additional economic value offered by EC as opposed to NC. Our data suggested that while EC incurred a cost of £48,964.66 with a QALY gain of 0.6992 per patient, NC incurred a cost of £55,531.29 for a QALY gain of 0.6540 per patient (Table 7). The ICER was then determined by dividing the difference in mean cost by the difference in mean outcome (QALYs), as shown below:

$$\mathrm{ICER }\left(\mathrm{\pounds }\right)=\frac{Cost \left(EC\right)-Cost \left(NC\right)}{QALY\left(EC\right)-QALY\left(NC\right)}=\frac{\Delta Cost}{\Delta QALY}=-\mathrm{\pounds 144,005}/\mathrm{QALY}$$

**Fig. 2:**
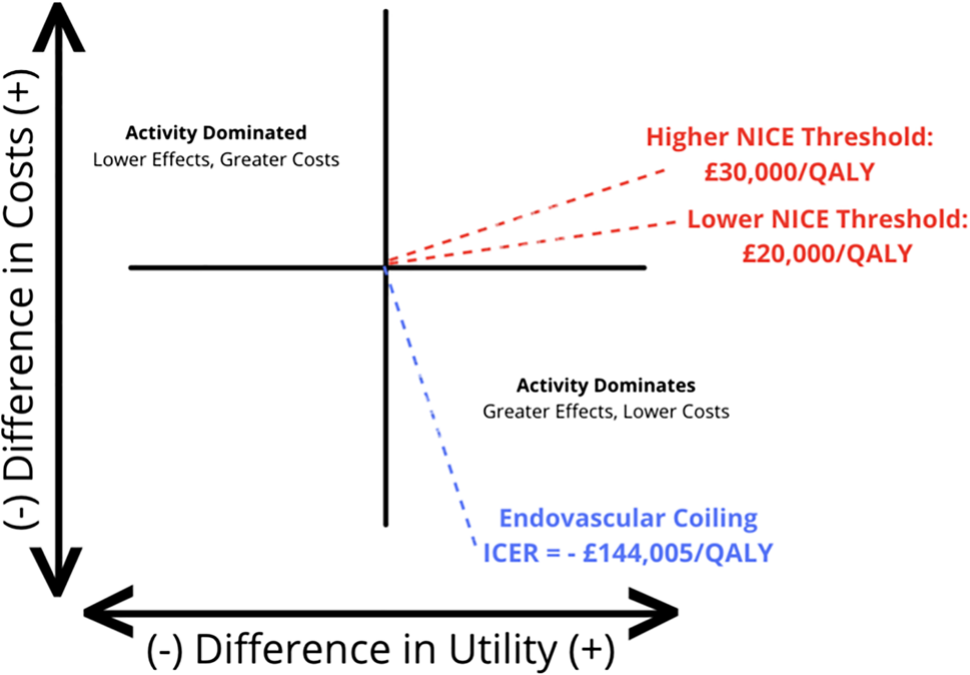
Cost-effectiveness plane. QALYs, quality-adjusted life years; NICE, National Institute for Clinical Excellence

This ICER indicates that for EC, every QALY gain would incur £144,005 less in comparison to NC. This would place EC in the lower right quadrant of the cost-effectiveness (CE) plane as seen in Fig. 2, signifying that this intervention offers improved health benefits at a lower cost. It is also less than NICE’s lower and upper willingness-to-pay (WTP) thresholds of £20,000 to £30,000 [20], reinforcing EC’s status as a more cost-effective intervention for wider adoption in the NHS.

### Monetary net benefit

The MNB expresses the additional economic value of an intervention versus its comparator in monetary units. It is calculated by multiplying the gained health benefit (QALYs) from the intervention with a chosen WTP threshold, and then subtracting the difference in cost incurred for this gained health benefit (as seen below).

$$\mathrm{MNB }(\mathrm{\pounds })=(WTP\times \Delta QALY)-\Delta Cost$$

We found an MNB of £7478.63 at the lower £20,000 threshold, and an MNB of £7934.63 at the upper £30,000 threshold (Table 8). Since the MNB is positive (> 0) for both, EC is considered a cost-effective intervention as compared to NC.

### Health net benefit

The health net benefit, typically expressed in QALYs, expresses the added health benefits of introducing a new intervention. It is calculated by first dividing the extra costs incurred by the WTP threshold and subtracting this from the overall gained QALYs (as seen below).

$$\mathrm{HNB }(\mathrm{QALY})=\Delta QALY-\frac{\Delta Cost}{WTP}$$

We found an HNB of 0.374 QALYs at the lower £20,000 threshold, and an HNB of 0.264 at the higher £30,000 threshold (Table 9). Since the HNB is positive (> 0) for both thresholds, EC is considered to have a positive net health benefit to the population, if EC were to be utilised instead of NC.

### mRS costs

A higher mRS score is associated with increased total costs due to greater treatment needs, including stroke unit care, intravenous thrombolysis and thrombectomy. The difference between mRS 1 and 2 (*p* = 0.0043), mRS 2 and 3 (*p* = 0.0007), mRS 3 and 4 (*p* = 0.0002), and mRS 3 and 5 (*p* = 0.0049) were all statistically significant. However, they were not significant between mRS 0 and 1 (*p* = 0.11) and mRS 4 and 5 (*p* = 0.82).

## Discussion

The ICER calculated demonstrates that EC would be a cost-effective intervention in comparison to NC. It is less than both NICE WTP thresholds [20] and belongs in the bottom right quadrant of the CE plane, as it offers greater health benefits for a lesser cost. Both MNB and HNB calculations are positive for both NICE thresholds as well, further reinforcing the idea that there is a substantial added monetary and health benefit in adopting EC over NC.

These results may be explained by inherent advantages of EC over NC in terms of the expected risks and benefits that each treatment can present with, which would therefore affect the utility of each procedure thereafter. In a study that compared the multiple English studies covering a total of 8836 patients who underwent EC and 7294 patients who underwent NC, it was found that the NC patients had lower mortality, lower chances of re-bleeding and re-treatments [7]. However, EC patients had significantly less post-operative complications and required less rehabilitation, with more favourable mRS scores overall [7]. These findings support an increased likelihood for EC patients to experience more favourable utility outcomes over NC patients. In the context of the UK, it was found that there was no significant difference in costs after 12 months between EC and NC patients [21], although the intervention cost for NC was slightly more at £34,714 while EC cost £30,431 [21] with the treatment cost for each resulting mRS state being consistent for both. Hence, with EC giving rise to a higher expected utility (EU) in QALYs combined with a lower expected cost (EC), EC can be seen to be more cost-effective than NC.

### Sensitivity analysis

Our utility data was obtained from a systematic review by Wolstenholme et al. which provided the health utility weighting in the form of EQ-5D stratified by the mRS [9]. For our sensitivity analysis, we sought to identify the effect of using another health utility scale on the ICER. The other health utility measure was the neuro-QoL which is bespoke for evaluating the quality of life in neurological conditions such as aSAH. This utility weighting was obtained from the same study that provided us with the EQ-5D utilities [9], and was used to calculate the new QALYs and subsequent ICER (Table 10).

The ICER value using the neuro-QoL utility measure was − £231,214 per QALY (a decrease of ≈£87,840 from the EQ-5D utility scale). Both utility measures consistently provide an ICER value in the southeast region of the incremental quadrant plot. While the ICER of both utility measures indicates that EC provides greater benefit at lower costs compared to NC, it is important to note that the higher recurrence rates associated with EC [22] may affect the ICER due to the increased costs incurred over a time horizon longer than 1 year. Ultimately, while the use of different health utility measures to calculate the ICER showed degrees of variation, they both reached the same conclusion of EC being more effective and less costly than NC.

### Study limitations

The first limitation of this CUA is found in the nature of aneurysmal SAH, which typically affects individuals between 50 and 55 years of age [23]. This was reflected in the ISAT trial, whereby the mean age in both the treatment groups was 52 years old [16]. However, the health utility weighting of the mRS we used was obtained from a systematic review and meta-analysis including studies on ischaemic stroke, haemorrhagic stroke, transient ischaemic stroke and subarachnoid haemorrhages [9]. The epidemiology of these conditions differs slightly, and thus using health utility weightings from this study may not be representative of the aneurysmal SAH cohort. This was corroborated in the same study which concluded that cohort-specific characteristics can influence mRS utility weighting [9]. This may have affected the economic evaluation as different utilities will affect the ICER value obtained.

A second limitation is the ambiguity surrounding the longer-term outcomes for EC; although the initial trial suggested more favourable 12-month outcomes, long-term follow-up found higher recurrence rates with EC [22]. Furthermore, a large meta-analysis from John Hopkins University found no consensus on the superiority of one treatment over the other [24]. In addition to this, costs used in this study only included the intervention cost and follow-up costs for the first 12-month window. Post-operative follow-up costs included the cost of complications such as re-rupture and vasospasm; however, data for the period after the initial 12 months was not obtained. As a result, the longer-term effectiveness of one intervention over the other is less comparable.

A third limitation lies in one of our data sources, where we obtained the costs per mRS health state over the first 12 months [12]. The study we referenced found the original cost data from another study conducted in Belgium with a sample size of 569 stroke patients [13]. Compared to the ISAT that studied 2143 patients, this population size is much smaller and only uses patients from hospitals in and around Belgium, which could therefore make the cost data less relevant to our UK-based study. Ideally, we would have used cost data from mainly the UK and Europe, similar to where the patients in the ISAT were from, if it were available.

Finally, the precision of the selection criteria of ISAT has been subject to criticism of selection bias. Of the 9559 patients with aSAH assessed for eligibility, 7416 of them were excluded due to contraindications for either of the two treatments [16]. Furthermore, the location of aneurysm influenced the treatment option in ISAT, as posterior circulation aneurysms were more likely to be allocated to coiling, whereas wide-necked aneurysms were more likely to be allocated to clipping [25]. These selection factors may have implications on the generalisability of the results.

### Literature review and contribution to current literature

A literature review was conducted using a combination of keywords ‘Endovascular Coiling’, ‘Neurosurgical Clipping’, ‘Aneurysmal Subarachnoid Haemorrhage’, ‘Cost Utility Analysis’ and ‘Economic Evaluation’ in 5 main databases—Embase, Medline, Google Scholar, Science Direct, PubMed. There were a few cost-effectiveness studies published by region or specific location, such as in developing countries [26] or the Republic of Korea [27], but no cost-utility analysis between both interventions has previously been performed. The search also yielded several systematic reviews and meta-analyses on the clinical outcomes and costs of both interventions. Though costs seemed to vary by region, most studies agreed that EC consistently demonstrated better outcomes, with reduced adverse effects [28], mortality and dependency in comparison to NC [29]. Out of 20 related articles that were identified, we focused on two key studies that provided us with the data needed to carry out our cost-utility analysis.

The International Subarachnoid Aneurysm Trial (ISAT) (*n* = 2143) is the sole large-scale randomised control trial comparing the adoption of EC to NC in the treatment of acute subarachnoid haemorrhage caused by aneurysmal rupture [3]. The study enrolled 2143 participants, all of which suffered aneurysmal rupture considered treatable with either of the two surgical interventions [16]. Results from the trial corroborate those of alternative studies [7, 8, 26] comparing EC to NC in the treatment of aSAH; EC was associated with an absolute reduction of 7.4% and a relative reduction of 23.9% in death and dependency at 12 months [16]. It should be acknowledged that since the publishing of the ISAT trial in 2002, there have been significant advances in several aspects of both procedures that can have a direct impact on patient outcomes, such as improvements in coil/catheter performance [30].

Wolstenholme and colleagues aimed to assess the findings elicited from the ISAT trial to evaluate the costs and resources used for each intervention. Data used was based on a sample of participants involved in the ISAT trial [21]. The study found that EC incurred higher costs than NC in terms of the cost of the surgery itself as well as any subsequent procedures [21]. This was more than offset, however, by lower costs associated with length of stay following the first episode of care as well as fewer costs related to complications and adverse events in the first 12 months [21]. However, these costs do not include the consumption of long-term nursing and informal care, both of which could incur significant costs [21].

Our findings can contribute to the current literature by providing the first CUA done in the UK, from the perspective of the NHS. While our study aligns with most of the relevant literature in terms of EC producing better clinical outcomes than NC, there is more variation in relative costliness of the two interventions according to region. In a CEA done in the USA, EC is instead found to be more expensive than NC, making the ICER positive (greater benefit at greater cost) and thereby exceeding the NICE thresholds and making it less cost-effective [19]. Another CEA performed in the developing country Pakistan also showed EC to incur higher costs than NC, while also claiming that both treatments did not produce significantly different clinical outcomes [26]. The variation in literature can be attributed to differences in geography as well as different materials and methods used. For example, the CEA in Pakistan had an analytic horizon of 6 months [26], while the CEA done in the USA also included additional costs that our CUA did not, such as the cost of cerebral angiography and/or rebleeding [19].

### Scope for further study and analysis

The use of the mRS score as an outcome measure in this study highlights the need for a holistic approach to cost evaluation for stroke patients; services such as social care and long-term nursing play a major role in the treatment of patients following the initial intervention and could incur significant costs to the healthcare system [31] (Table 11). Further research should endeavour to account for these costs alongside any potential costs associated with productivity loss. As previously acknowledged, there have also been significant advances in the performance of both procedures since the year the cost data was obtained [30]. It would therefore be beneficial to account for these improvements in future research, providing a more up-to-date cost breakdown for each intervention.

Furthermore, the disparity in cost-effectiveness between EC and NC should not be the sole differentiating factor in deciding which treatment would be most suitable for every patient, as there are also individual patient and aneurysm-related considerations to note. For example, while the EQ-5D questionnaire asks about certain symptoms to assess health-related quality of life, there may be inconsistencies in which symptoms patients value the most, with the possibility of many of these unforeseen symptoms being unaccounted for. Future studies could incorporate additional utility scales other than the EQ-5D and neuro-QoL that we used in this study, to account for more patient-specific factors that affect the perceived utility of each treatment. Moreover, our analysis only investigated the difference in cost-effectiveness in using these treatments specifically for an aSAH, while EC and NC can also be used in other aneurysm-related conditions such as a subdural haematoma—whereby differences in treatment utility and outcomes may not be the same. Thus, the type of aneurysm suffered should also be factored in when choosing EC or NC for a patient, and future studies could be done to determine if EC remains to be more cost-effective than NC for similar conditions to aSAH.

## Conclusion

Both EC and NC are viable and widely utilised treatment options for aSAH resulting from aneurysmal rupture [8]. Considering the costs of both the intervention and complications relative to the associated utilities for each procedure, EC was found to be more cost-effective than NC, with an ICER value of − £144,005 (below both NICE WTP thresholds) alongside positive MNB and HNB values. EC is also associated with lower incidences of death and dependency in the first year following the procedure, despite a slightly higher risk of rebleeding [16]. The findings of this report add to current literature supporting the increasing preference for EC for the treatment of aSAH in the UK [18]. Clinical and financial benefits of the intervention align well with core NHS principles for the provision of the ‘best value for taxpayers’ money’ and ‘care that is effective and focused on patient experience’ [32].

## Data Availability

Data is available on reasonable request to the corresponding author.

## Appendix

**Table 1:** Table describing each mRS health state

Score	Description
mRS 0	No symptoms at all
mRS 1	No significant disability despite symptoms; able to carry out all usual duties and activities
mRS 2	Slight disability; unable to carry out all previous activities, but able to look after own affairs without assistance
mRS 3	Moderate disability; requiring some help, but able to walk without assistance
mRS 4	Moderately severe disability; unable to walk without assistance and unable to attend to own bodily needs without assistance
mRS 5	Severe disability; bedridden, incontinent and requiring constant nursing care and attention
mRS 6	Dead

Data taken from [33]. *mRS*, modified Rankin scale.

**Table 2:** Calculation of the 2020/2021 price for endovascular coiling

	Mean cost per patient in £s (2003/2004)	Discount rate (1.035^17^)	Discounted mean cost per patient in £s (2020/2021)
Total cost of intervention (first episode of care)	4520	1.795	8113.400
Total cost of imaging and investigations	886	1.795	1590.3700
Total cost of stay (first episode of care)	11,547	1.795	20,726.865
Total cost per patient	16,953	1.795	30,430.635

2003/2004 costs taken from [21].

**Table 3:** Calculation of the 2020/2021 price for neurosurgical clipping

	Mean cost per patient in £s (2003/2004)	Discount rate (1.035^17^)	Discounted mean cost per patient in £s (2020/2021)
Total cost of intervention (first episode of care)	3146	1.795	5647.07
Total cost of imaging and investigations	882	1.795	1583.19
Total cost of stay (first episode of care)	15,311	1.795	27,483.245
Total cost per patient	19,339	1.795	34,713.505

2003/2004 costs taken from [21].

**Table 4:** Cost associated with each mRS state [29]

mRS score	Cost in £s (2016/2017)	Discount rate (1.035^4^)	Discounted cost in £s (2020/2021)
0	6620	1.148	7599.760
1	11,196	1.148	12,853.008
2	18,929	1.148	21,730.492
3	35,771	1.148	41,065.108
4	60,118	1.148	69,015.464
5	60,458	1.148	69,405.784
6	0	1.148	0

2017/2017 costs taken from [12].

**Table 5:** Probabilities and utilities assigned to each mRS state

mRS score	EQ-5D utility weighting	Probability of outcome	
		Endovascular coiling	Neurosurgical clipping
0	0.93	0.245	0.177
1	0.86	0.283	0.277
2	0.68	0.237	0.237
3	0.56	0.101	0.134
4	0.31	0.028	0.040
5	0.06	0.026	0.036
6	0	0.08	0.099

EQ-5D utility weightings taken from [9]. *mRS*, modified Rankin scale.

Outcome probabilities taken from [16].

**Table 6:** Decision tree values and calculations

	Outcome	Expected utility (EU) in QALYs	Calculation	Expected cost (EC) in £s	Calculation
Endovascular coiling	A	0.2279	EU = 0.93 × 0.245	9317.54	EC = (30,431 + 7599.76) × 0.245
	B	0.2434	EU = 0.86 × 0.283	12,249.37	EC = (30,431 + 12,853.00) × 0.283
	C	0.1612	EU = 0.68 × 0.237	12,362.27	EC = (30,431 + 21,730.49) × 0.237
	D	0.0566	EU = 0.56 × 0.101	7221.11	EC = (30,431 + 41,065.11) × 0.101
	E	0.0087	EU = 0.31 × 0.028	2784.50	EC = (30,431 + 69,015.46) × 0.028
	F	0.0016	EU = 0.06 × 0.026	2595.76	EC = (30,431 + 69,405.78) × 0.026
	G	0.0000	EU = 0.00 × 0.08	2434.48	EC = (30,431 + 0.00) × 0.08
	Node 2	0.6992	EU = 0.2279 + 0.2434 + 0.1612 + 0.0566 + 0.0087 + 0.0016 + 0.0000	**48,965.03**	EC = 9317.54 + 12,249.37 + 12,362.27 + 7221.11 + 2784.50 + 2595.76 + 2434.48
Neurosurgical clipping	H	0.1646	EU = 0.93 × 0.177	7489.54	EC = (34,714 + 7599.76) × 0.177
	I	0.2380	EU = 0.86 × 0.277	13,176.06	EC = (34,714 + 12,853.00) × 0.277
	J	0.1612	EU = 0.68 × 0.237	13,377.34	EC = (34,714 + 21,730.49) × 0.134
	K	0.0750	EU = 0.56 × 0.134	10,154.40	EC = (34,714 + 41,065.11) × 0.134
	L	0.0124	EU = 0.31 × 0.04	4149.18	EC = (34,714 + 69,015.46) × 0.04
	M	0.0022	EU = 0.06 × 0.036	3748.31	EC = (34,714 + 69,405.78) × 0.036
	N	0.000	EU = 0.00 × 0.99	3436.69	EC = (34,714 + 0.00) × 0.099
	Node 3	0.6534	EU = 0.1646 + 0.2380 + 0.1612 + 0.0750 + 0.0124 + 0.0022 + 0.0000	**55,531.52**	EC = 7489.54 + 13,176.06 + 13,377.34 + 10,154.40 + 4149.18 + 3748.31 + 3436.69

**Table 7:** Costs and QALYs of both interventions over 1 year

Intervention	Discounted mean cost per patient in £s (2020/2021)	QALYs gained per patient
Endovascular coiling	48,964.66	0.6992
Neurosurgical clipping	55,531.29	0.6540
$\Delta$	− 6566.63	0.0452

*QALYs*, quality-adjusted life years.

**Table 8:** Monetary net benefit (MNB) data

	NICE lower vs. upper WTP thresholds	
Willingness-to-pay (£)	£20,000	£30,000
$\Delta QALY$	0.0452	0.0452
$\Delta Cost$(£)	− 6566.63	− 6566.63
MNB (£)	7478.63	7934.63

*MNB*, monetary net benefit; *QALY*, quality-adjusted life year.

**Table 9:** Health net benefit (HNB) data

	NICE lower vs. upper WTP thresholds	
Willingness-to-pay (£)	£20,000	£30,000
$\Delta QALY$	0.0452	0.0452
$\Delta Cost$(£)	− 6566.63	− 6566.63
HNB (QALY)	0.374	0.264

*HNB*, health net benefit; *QALY*, quality-adjusted life year.

**Table 10:** Sensitivity analysis calculations

Sensitivity analysis	ICER (£ per QALY)	Δ ICER vs. standard (Δ £/QALY)
Standard (EQ-5D)	− 144,005.06	0
Utility: SF-36	− 171,897.64	− 28,524.50
Utility: neuro-QoL	− 231,214.44	− 87,841.30

*EQ-5D*, Euro-QoL 5-dimension; *ICER*, incremental cost-effectiveness ratio; *QALY*, quality-adjusted life year.

**Table 11:** Breakdown of costs for the two interventions (using 2003/2004 costs)

Cost category	Mean cost per patient in £s	
	Endovascular coiling	Neurosurgical clipping
Total cost staff	1450	2108
Total cost equipment	183	35
Total cost consumables	2627	901
Total cost further procedures before discharged	260	102
Total cost of intervention (first episode of care)	4520	3146
Total cost of imaging and investigations (first episode of care)	886	882
Number of days in ITU	2872	3573
Number of ward days	5215	6103
Number of DGH days	2371	3346
Number of rehabilitation clinic days	1089	2289
Total cost of length of stay (first episode of care)	11,547	15,311
Total cost per patient	16,953	19,339

Intervention costs taken from (21).

## Code availability

Code is available on reasonable request to the corresponding author.
